# Comparison of ADC metrics and their association with outcome for patients with newly diagnosed glioblastoma being treated with radiation therapy, temozolomide, erlotinib and bevacizumab

**DOI:** 10.1007/s11060-014-1636-6

**Published:** 2014-10-29

**Authors:** Qiuting Wen, Laleh Jalilian, Janine M. Lupo, Annette M. Molinaro, Susan M. Chang, Jennifer Clarke, Michael Prados, Sarah J. Nelson

**Affiliations:** 1 Department of Radiology and Biomedical Imaging, University of California, San Francisco (UCSF), 1700 4th Street, Byers Hall, Box 0775, San Francisco, CA 94143-0775 USA; 2 UCSF/UCB Joint Graduate Group in Bioengineering, University of California, San Francisco (UCSF), San Francisco, CA USA; 3 Department of Bioengineering and Therapeutic Sciences, University of California, San Francisco (UCSF), San Francisco, CA USA; 4 Department of Neurological Surgery, University of California, San Francisco (UCSF), San Francisco, CA USA

**Keywords:** Newly diagnosed GBM, ADC, Bevacizumab, Anti-angiogenic agent

## Abstract

To evaluate metrics that describe changes in apparent diffusion coefficient (ADC) and to examine their association with clinical outcome for patients with newly diagnosed GBM who were participating in a Phase II clinical trial of treatment with radiation (RT), temozolomide, erlatonib and bevacizumab. Thirty six patients were imaged after surgery but prior to therapy and at regular follow-up time points. The following ADC metrics were evaluated: (1) histogram percentiles within the T2-hyperintense lesion (T2L) at serial follow-ups; (2) parameters obtained by fitting a two-mixture normal distribution to the histogram within the contrast-enhancing lesion (CEL) at baseline; (3) parameters obtained using both traditional and graded functional diffusion maps within the CEL and T2L. Cox Proportional Hazards models were employed to assess the association of the ADC parameters with overall survival (OS) and progression-free survival (PFS). A lower ADC percentile value within the T2L at early follow-up time points was associated with worse outcome. Of particular interest is that, even when adjusting for clinical prognostic factors, the ADC_10%_ within the T2L at 2 months was strongly associated with OS (*p* < 0.001) and PFS (*p* < 0.007). fDM metrics showed an association with OS and PFS within the CEL when considered by univariate analysis, but not in the T2L. Our study emphasizes the value of ADC metrics obtained from the T2L at the post-RT time point as non-invasive biomarkers for assessing residual tumor in patients with newly diagnosed GBM being treated with combination therapy that includes the anti-angiogenic agent bevacizumab.

## Introduction

Bevacizumab is a humanized monoclonal VEGF-blocking antibody that has been shown to normalize vascular permeability and regulate angiogenesis in patients with glioblastoma (GBM). Although it has been shown to reduce the volume of the contrast enhancing lesion (CEL) on post-Gadolinium T1-weighted MR images and to provide improved time to progression in patients with recurrent disease [1–3], recent Phase II and Phase III clinical trials indicated that it is ineffective at extending overall survival for patients with newly diagnosed GBM [4–7]. With a growing number of studies providing evidence for increased tumor invasiveness following treatment failure in patients receiving bevacizumab [8], it is important to identify at an early stage which patients are benefiting from anti-angiogenic therapies, as opposed to treating all patients in the same manner. Monitoring the effectiveness of bevacizumab is challenging using conventional measures of response to therapy because reductions in the CEL may be due to an anti-permeability effect rather than a reduction in bulk tumor [9], which is commonly referred to as “pseudoresponse” [10, 11]. Differentiation of non-enhancing tumor within the T2L from edema or gliosis is important for effectively monitoring response to bevacizumab and similar anti-angiogenic agents.

The apparent diffusion coefficient (ADC) is a metric that characterizes the random motion of water molecule protons at a microscopic level and may provide valuable insights to tumor physiology. Decreases in ADC have been proposed as a non-invasive measure of tumor cellularity and increases in ADC to reflect a breakdown of tissue architecture [12–16]. A number of different strategies have been proposed to define metrics in predicting clinical outcome and monitoring response to therapy following treatment with bevacizumab. These include parameters derived from the histogram of ADC values within the anatomic lesion at a single time point [17–19], and from functional diffusion maps (fDMs) that evaluate serial changes in ADC on a pixel by pixel basis [20–26]. For patients with recurrent GBM being treated with bevacizumab, low values in the pretreatment ADC histogram from the CEL that were fit to a two normal distribution mixture curve were found to be associated with poor outcome [17, 18], but in the up-front setting low ADC was found to be associated with significantly longer PFS [19]. When fDM analysis was used in patients with recurrent GBM [25, 26], prior studies showed that the volumes of tissue within the CEL and T2L that had reduced ADC values between baseline and early post-treatment scans were associated with PFS and OS.

Although these initial results indicate that ADC metrics may be helpful in predicting treatment effectiveness for patients with recurrent GBM, their utility has not yet been fully explored for combination treatments that are being applied in an upfront setting. Obtaining a detailed understanding of how to interpret early changes in these parameters and integrate them into criteria used for assessing treatment response could have a significant impact on patient care. The purpose of this study was to evaluate the association of ADC metrics with clinical outcomes for patients with newly diagnosed GBM who were participating in a Phase II clinical trial that included bevacizumab.

## Materials and methods

### Patient population

A total of 151 MR scans that include diffusion weighted imaging (DWI) were obtained from 36 patients with newly diagnosed GBM who were participating in a Phase II clinical trial during the period between January 9, 2009 and April 3, 2012 (29 scans at baseline, 25 patients had complete serial scans from baseline until progression). All patients had pathologically confirmed GBM, a Karnofsky Performance Score (KPS) of at least 60 and had undergone prior biopsy (five patients) or surgical resection (10 gross-total and 21 sub-total) but no other prior therapy. Patient age ranged from 21 to 76 years, with a median of 52 years. Treatment included external beam radiation therapy to an average dose of 60 Gy and was delivered to the tumor site in 2-Gy fractions over a 6-week period. The protocol called for temozolomide to be given at a daily dose of 75 mg/m^2^, during radiation therapy and at 200 mg/m^2^ for 5 days every 28 days afterwards, for erlotinib to be given daily both during and after radiation, and for bevacizumab to be given at a dose of 10 mg/kg IV every 2 weeks, starting at approximately 2 weeks into radiation therapy [5]. All patients participating in this study gave informed consent according to the guidelines of our institutional review board. Progression was determined based on the recently defined RANO criteria [10].

### MR imaging and post-processing

All scans were obtained using a 3T GE MR scanner. Time points selected for study were at baseline (post-surgical resection and prior to therapy), 1 month (mid-RT), 2 months (post-RT) and every 2 months thereafter until presumed tumor progression (up to a maximum of 14 months). Standard anatomical MR imaging included axial T2-weighted fluid attenuated inversion recovery (FLAIR) images and pre- and post-contrast T1-weighted spoiled gradient echo (SPGR). DWI were acquired with b = 1,000 (dir = 6, NEX = 4) and ADC maps were calculated using in-house developed software. CEL regions were manually defined on the coregistered post-contrast T1 SPGR images at each available time point. Any hyperintense signal that was also present on the pre-contrast T1 images was assumed to be indicative of acute blood products and was excluded. The T2L regions were segmented based on the hyper-intensity region of FLAIR images using a semi-automatic region-growing segmentation tool [27]. The resection cavity was excluded from all ROIs.

#### Histogram analysis within the T2L and CEL

**Fig. 1:**
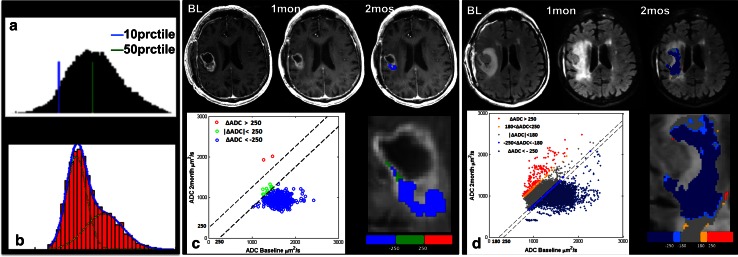
Illustration of methods for analyzing ADC: **a** Percentile values extracted from the histogram of ADC values in the T2L. **b** 2-mixture normal distribution fitting on ADC histograms in CEL. **c** Traditional fDMs within the CEL overlaid on a T1 post-contrast image at 2 months with scatter plot of the distribution of ADC changes for the entire CEL. **d** Graded fDMs within the T2L overlaid on a FLAIR image at 2 months with scatter plot of the distribution of ADC changes for the entire T2L

In regions of interest corresponding to T2Ls at time points up to 8 months after the start of treatment, ADC histograms followed an approximately normal distribution and were characterized using percentile values (Fig. 1a). In this case the 10th and 50th percentiles were chosen for subsequent analysis to represent regions with more aggressive tumor. At baseline, histograms of the ADC within the CEL were also fit with a 2-mixture normal distribution (Fig. 1b). Mean values for the lower peak (ADC_L_) and the lower curve proportion (LCP) were calculated in the manner proposed by Pope et al. [17].

#### Functional diffusion map

For fDM analysis, ADC maps at baseline and 2 months were co-registered using an affine registration with 12° of freedom to ensure adequate alignment (http://www.fmrib.ox.ac.uk/fsl/). Voxel-wise subtraction was performed between 2 months and baseline ADC maps. Both traditional fDMs (Fig. 1c) [20] and graded fDMs (Fig. 1d) [25] were generated. Due to the fact that our data were acquired at a field strength of 3T than 1.5T as was used for these earlier studies, a set of new thresholds were generated in the same way as described in the literature [21, 24, 25]. For each patient, the volume of tissue showing decreased ADC (Vol_ΔADC_), as well as the normalized volume showing decreased ADC within the CEL and T2L (%Vol_ΔADC_, which was normalized against the overlapping lesion volume), were calculated.

### Statistical analysis

Both univariate and multivariate Cox Proportional Hazards (CoxPH) model with covariates of baseline KPS, age, and extent of resection (0-biopsy, 1-subtotal, 2-grosstotal) were employed to evaluate the relationship of the fitted parameters to progression-free survival (PFS) and overall survival (OS), landmarked from the scan date of the diffusion parameters. In the case of no progression or death, the event time was censored at the date of last contact. Classification and regression tree (CART) analysis was utilized to determine the cut-off for dichotomizing the fitted parameters [28]. Kaplan–Meier survival curves for each subgroup determined by the CART split points were compared using a log-rank test. Owing to the exploratory nature of the study, no formal adjustment of type I error was undertaken. In all cases, *p* < 0.05 was considered statistically significant (Matlab 2012a).

## Results

### Clinical

Median OS was 86.1 weeks with nine patients censored and median PFS was 56.1 weeks with four patients censored for the 36 patients considered in this analysis, which is consistent with our recent report on a larger population study [5]. At the time of progression, 23 patients had enhancing progressive disease, 11 patients had non-enhancing progressive disease with only enlarged FLAIR lesion volume, and two patients died before imaging follow-up. Of the baseline clinical factors (KPS, age, gender and extent of resection), only the extent of resection was significantly associated with OS (Univariate, *p* < 0.002, HR = 0.285, 95 % CI = 0.134–0.608) and PFS (Univariate, *p* < 0.006, HR = 0.366, 95 % CI = 0.179–0.748).

### Volumes of anatomic lesions

Table 1 lists the median and range of T2L and CEL volumes at different time points. There was a noticeable reduction at 1 and 2 months in the volumes of both CEL and T2L. When considered as single variable, the volumes of the CEL at 1 and 2 months were associated with OS (*p* < 0.003, HR = 1.22 at 1 month; *p* < 0.03, HR = 1.37 at 2 months) and PFS (*p* < 0.03, HR = 1.11 at 1 month; *p* < 0.02, HR = 1.38 at 2 months). When adjusted for clinical factors these associations were no longer significant. The volumes of the T2L were not associated with OS or PFS.

**Table 1:** Volume for Anatomic Lesions [median (min–max) in cc]

	Baseline	1 month	2 months	4 months	6 months	8 months
T2L	30.07 (1.71–142.60)	23.52 (1.51–140.42)	9.55 (0.05–41.64)	10.64 (0.28–43.55)	13.72 (0.37–46.35)	14.93 (0.12–44.17)
CEL	3.12 (0.19–21.94)	1.22 (0–17.65)	1.08 (0–7.2)	0.08 (0–3.13)	0.12 (0–1.93)	0.03 (0–4.02)

### Histogram analysis

**Fig. 2:**
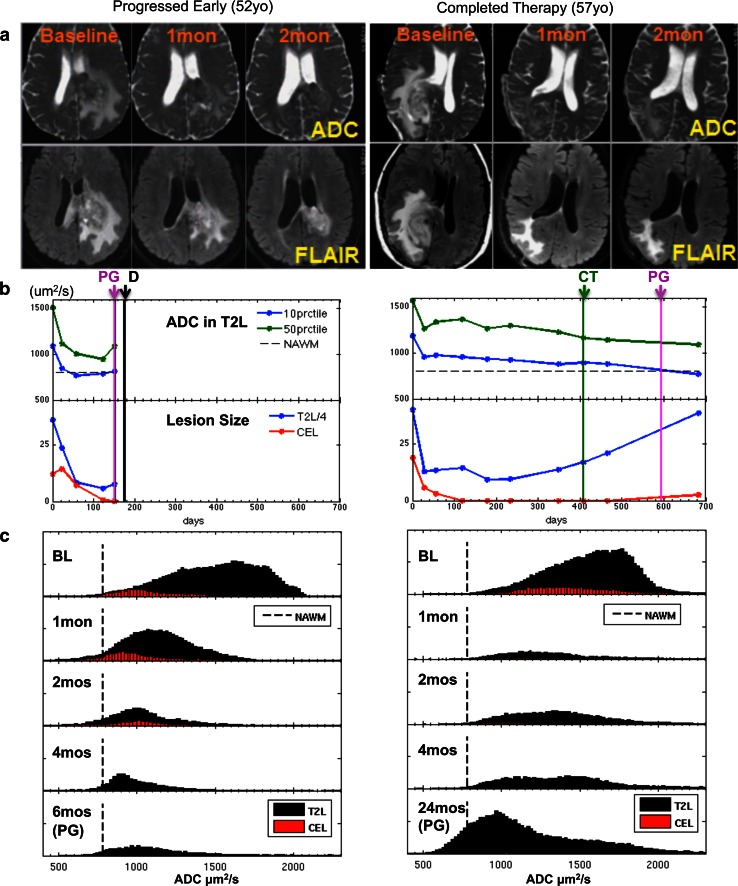
Comparison of serial displays for two patients (*left*—progressed early, *right*—completed therapy without signs of progression) who both had large T2L at baseline. T2L and ADC were significantly reduced in both patients immediately following onset of therapy. At post-RT, residual T2Ls were comparable for both patients, but ADC percentage values were much lower in the patient who progressed early than the patient who was stable. **a** ADC and FLAIR images at baseline, 1 and 2 months. **b** Serial display of ADC percentiles and lesion sizes. (*PG* progression, *CT* completed therapy, *D* deceased.). **c** Serial display of ADC histograms in T2L and CEL lesions

Within the T2L, the CoxPH model coefficients showed a significant association for values of ADC_10%_ and ADC_50%_ with OS and PFS (Table 2). A lower ADC percentile value within the T2L indicated a poorer prognosis. The ADC_10%_ at 2 months (post-RT) was associated with PFS (univariate CoxPH, *p* < 0.03, HR = 0.52, 95 % CI = 0.29–0.93) and OS (univariate CoxPH, *p* < 0.01, HR = 0.37, 95 % CI = 0.18–0.79). Adjusting for baseline KPS, age, extent of resection, Cox regression analysis confirmed that lower ADC_10%_ within T2L at 2 months is still a risk factor for OS (multivariate CoxPH, *p* < 0.001, HR = 0.11, 95 % CI = 0.03–0.41) and PFS (multivariate CoxPH, *p* < 0.007, HR = 0.31, 95 % CI = 0.13–0.72). Serial ADC percentile changes of two age-matched patients who both had large T2L at baseline are shown in Fig. 2. One patient progressed early and the other was stable and completed therapy after being on treatment for 12 months. T2L and ADC were significantly reduced in both cases immediately following onset of therapy (Fig. 2a). At post-RT, T2L volumes were comparable for both patients, but ADC percentage values were much lower in the patient who progressed early than the patient who was stable (Fig. 2b). Figure 2c shows profiles of ADC histograms within T2L and CEL over time.

**Table 2:** Summary of multivariate CoxPH results with adjustment for KPS, age and extent of resection

Type	Parameters considered	Time point	OS		PFS	
			*p* value	HR	*p* value	HR
Lesion size	Vol_CEL_ (cc)	BL	0.315	0.95 [0.87 1.05]	0.900	0.99 [0.92 1.08]
		2mos	0.301	1.23 [0.83 1.81]	0.201	1.26 [0.89 1.79]
	Vol_T2L_ (cc)	BL	0.431	0.99 [0.98 1.01]	0.837	1.00 [0.99 1.15]
		2mos	0.326	1.03 [0.97 1.10]	0.421	1.02 [0.97 1.07]
Histogram— 2-mixture normal fitting	ADC_L_	BL	0.91	1.01 [0.80 1.29]	0.75	0.95 [0.79 1.18]
Histogram—Percentiles in T2L	ADC_10%_ (μm^2^/s/100)	BL	0.116	0.77 [0.55 1.07]	0.032*	0.69 [0.50 0.97]
		1mos	0.188	0.54 [0.21 1.36]	0.014*	0.39 [0.18 0.83]
		2mos	0.001*	0.11 [0.03 0.41]	0.007*	0.31 [0.13 0.72]
		4mos	0.005*	0.43 [0.23 0.78]	0.024*	0.54 [0.32 0.92]
	ADC_50%_	4mos	0.011*	0.61 [0.42 0.89]	0.045*	0.73 [0.54 0.99]
Traditional fDM in T2L	Vol_ΔADC<−250_ (cc)	BL-1mos	0.927	0.99 [0.82 1.20]	0.963	1.00 [0.84 1.20]
		BL-2mos	0.552	1.13 [0.75 1.70]	0.603	1.09 [0.78 1.54]
Graded fDM in T2L	Vol_250<ΔADC<−180_	BL-1mos	0.489	0.486 [0.06 3.76]	0.577	0.63 [0.13 3.16]
		BL-2mos	0.347	2.36 [0.39 14.19]	0.481	1.65 [0.41 6.71]

*p < 0.05

**Fig. 3:**
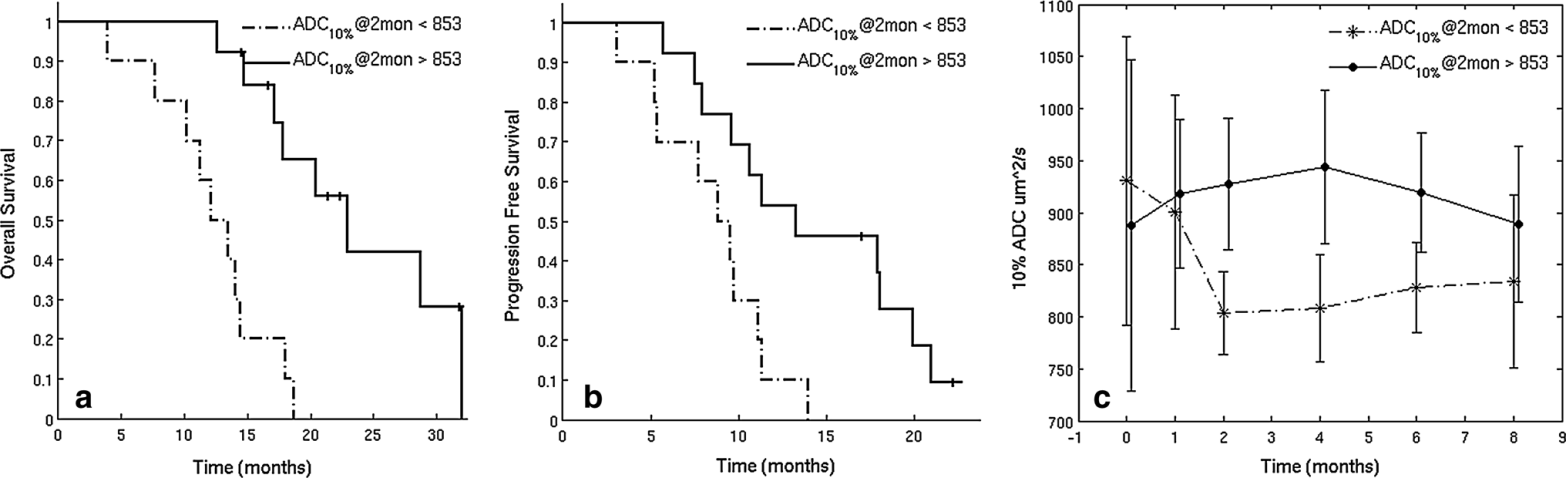
Stratification of patients based on CART analysis of ADC_10%_ in T2L at 2 months. **a** Kaplan–Meier curves for each group when split on CART threshold at 2 months for OS with ADC_10%_ < 853 µm^2^/s in dash line (12 patients), ADC_10%_ >853 µm^2^/s in solid line (13 patients). **b** Kaplan–Meier curves for each group when split on CART threshold at 2 months for PFS with ADC_10%_ <853 µm^2^/s in dash line (12 patients), ADC_10%_ >853 µm^2^/s in solid line (13 patients). **c** The mean and standard deviation for ADC_10%_ over time for each CART split group

A cutoff value of 853 µm^2^/s at the 2 month time point was determined by CART analysis to differentiate patients into two groups based on OS (log-rank, *p* = 0.00048) (Fig. 3a), and a cutoff value of 853 µm^2^/s based on PFS (log-rank, *p* = 0.02) (Fig. 3b). The mean and standard deviation for ADC_10%_ over time for each split group is shown in Fig. 3c. At baseline, no parameters from the 2-mixture normal fitting were found to be associated with either OS or PFS (*p* > 0.1).

### fDM

The traditional fDM technique typically applies a single ΔADC threshold to classify voxels into increasing or decreasing ADC. The 95 % confidence interval for defining normal-appearing white and grey matter was 250 µm^2^/s for our protocol. For graded fDMs, the 95 % confidence interval for defining normal-appearing white matter was 180 µm^2^/s for our data set.

Within the CEL, the recommended minimum overlapping CEL volume that should be considered is 4 cc for traditional fDM [20]. In this study, due to the strong anti-angiogenic effect, none of our patients had an overlapping CEL larger than 4 cc. Without consideration of this criteria, the traditional fDM and graded fDM analyses within the CEL provided parameters that were associated with OS and PFS (Vol_ΔADC<−250_ with OS, *p* < 0.003; HR = 9.52; Vol_−250<ADC<−180_ with OS, *p* < 0.03, HR = 15509; with PFS *p* < 0.03, HR = 23775). However, these were not significant when adjusting for clinical factors. Within the T2L, none of these parameters were found to be associated with OS or PFS (Table 2).

## Discussion

Although bevacizumab has been shown to reduce the volume of the contrast enhancing and T2 lesions after the initiation of therapy, the highly variable response and limited improvement in OS times highlight the need for identifying alternative parameters that can more accurately predict treatment outcomes. Diffusion imaging techniques are dependent on the microscopic structure of tissue, and are sensitive to cell density and necrosis as well as vasogenic edema. It is for this reason that analysis of the ADC maps has been proposed as a method for providing information about the properties of both enhancing and non-enhancing tumor.

Consistent with previous report for patients treated with bevacizumab [1–4], there were reductions in the volumes of the CEL and T2L at 1 and 2 month follow-up scans (Table 1), and the CEL volume was associated with survival as a univariate variable [29]. However, this association was no longer significant when adjusting for clinical factors, suggesting that the CEL volume does not add value in addition to clinical factors in relation to survival. We would like to note that due to the strong anti-leakage effect of bevacizumab, over half patients demonstrated CEL volume <1 cc at 2 month, and 1/3 patients had non-enhancing progressive disease. All these motivated us to look more closely at the T2L as the region of interest for imaging biomarkers.

Regions within T2L with low ADC values are thought to correspond to regions of higher cellularity, while regions with increased ADC to correspond to vasogenic edema [32–34]. Both of these opposing effects are present within the tumor microenvironment and may therefore counteract each other. In tumors being actively treated with bevacizumab, vasogenic edema is more effectively controlled [30], resulting in a reduction in the volume of the T2L and lower ADC values that may more closely reflect the cellularity of the tumor. Our results support this hypothesis by indicating that lower ADC percentiles within the T2L at 2 months time window were significant risk factors for both PFS and OS. Two factors that could influence ADC values in the earlier and later time window and confound the interpretation of the data are ischemia that results from the surgical resection and RT-induced edema. Regions of ischemia occur around the resection cavity may result in temporarily reduced ADC values that typically return to normal within 90 days [31]. Regions of reduced ADC that are observed during this early time frame should therefore be interpreted with caution, as they may be confused with recurrent tumor. In the later time frame (e.g. post-4mon), increases in edema that occur during RT may result in higher ADC values, which could mask the presence of tumor. With bevacizumab, the confounding effects from surgery and during RT appear to have resolved at 2 month, so that the ADC values provided a more accurate representation of residual tumor. At subsequent time points, reactive edema associated with growing tumor may result in elevated ADC (Fig. 3c). Another potential confounding factor is gelatinous necrosis, which could cause persistent restricted diffusion in bevacizumab treated patients [35–37]. Caution must be exerted in interpreting restricted diffusion because it has been reported that patients who demonstrated such bevacizumab caused necrosis had longer survival [35]. The average time of detecting such necrosis with diffusion was 8 months, therefore it is unlikely to have developed by the 2 month follow-up in our study (6 weeks into bevcizumab). We hypothesize that it is for these reasons that the 2 months (post-RT) time point appeared to be the best time point for using ADC to assess residual tumor.

Although previous studies have shown that the two normal distribution mixture curve analysis of pre-surgery ADC histograms in the CEL can predict response to bevacizumab for patients with newly diagnosed GBM [19], we were unable to detect an association for our patient population. This may have been due to our baseline data having been post-surgery and therefore lacking information about the resected tumor and/or being influenced by surgically-induced ischemia.

The fDM analysis was developed to examine voxel-wise changes in ADC in the patient over time. Our results showed that the fDM analysis of higher volumes of tissue within the CEL that showed decreased ADC were associated with worse PFS and OS when considered without adjustment for clinical factors. While this may be a less sensitive metric than others, the finding is consistent with areas of reduced diffusion corresponding to more cellular tumor and hence inferring a worse outcome. The global reduction in ADC metrics that we observed within the T2L is likely to be due to reabsorption of edema after treatment.

Despite the promising results obtained in this study using fDM analysis, there are limitations that should be taken into account in patients treated with bevacizumab in the up-front setting. First, the CEL volumes of all patients in this study were smaller than the minimum recommended size (4 cc) to be considered for the traditional fDM [20]. A second limitation is in the accuracy of the image registration methods used to align serial ADC images. Significant tissue shifts were observed in some of our patients after initial of therapy, mainly because of the reduction in edema caused by the anti-angiogenic agent, which reduces the intracranial pressure. In these cases, accurate tissue matching between different time points can be challenging, even with non-linear registration.

In conclusion, our study emphasizes the value of ADC metrics for early assessment of residual tumor in patients with newly diagnosed GBM being treated with a combination of therapy that includes bevacizumab. While there was a rapid decline of ADC percentile values immediately following onset of therapy in almost all subjects, the ADC percentile values were lower for the patients who progressed early. This suggests that tracking the changes in ADC using serial histogram analysis as shown in Fig. 2 could potentially assist radiologists in monitoring patient response to therapy that includes bevacizumab. Our results highlighted the value of ADC_10%_ within the T2L at the post-RT exam in conjunction with standard clinical factors in predicting PFS and OS. We hypothesize that this is due to the anti-angiogenic effect of bevacizumab reducing the extent of vasogenic edema at this time point and therefore allowing the observed ADC values to more accurately reflect the residual tumor burden.
